# Validation of the Mayo Hip Score: construct validity, reliability and responsiveness to change

**DOI:** 10.1186/s12891-016-0868-3

**Published:** 2016-01-19

**Authors:** Jasvinder A. Singh, Cathy Schleck, W. Scott Harmsen, David G. Lewallen

**Affiliations:** Medicine Service, Birmingham VA Medical Center and Department of Medicine, University of Alabama, Faculty Office Tower 805B, 510 20th Street S, Birmingham, AL 35294 USA; Departments of Orthopedic Surgery, Mayo Clinic School of Medicine, Rochester, MN USA; Departments of Biostatistics, Mayo Clinic School of Medicine, Rochester, MN USA; Center for Surgical Medical Acute care Research and Transitions (C-SMART), Birmingham VA Medical Center, Birmingham, AL USA; Division of Epidemiology, School of Public Health, University of Alabama, Birmingham, AL USA

**Keywords:** Validation, Mayo hip score, Mayo hip questionnaire, Total hip arthroplasty, Total hip replacement, Validity, Responsiveness, Minimal clinically meaningful difference, MCID, Revision risk, Reliability

## Abstract

**Background:**

Previous studies have provided the initial evidence for construct validity and test-retest reliability of the Mayo Hip Score. Instruments used for Total Hip Arthroplasty (THA) outcomes assessment should be valid, reliable and responsive to change. Our main objective was to examine the responsiveness to change, association with subsequent revision and the construct validity of the Mayo hip score.

**Methods:**

Discriminant ability was assessed by calculating effect size (ES), standardized response mean (SRM) and Guyatt’s responsiveness index (GRI). Minimal clinically important difference (MCII) and moderate improvement thresholds were calculated. We assessed construct validity by examining association of scores with preoperative patient characteristics and correlation with Harris hip score, and assessed association of scores with the risk of subsequent revision.

**Results:**

Five thousand three hundred seven provided baseline data; of those with baseline data, 2,278 and 2,089 (39 %) provided 2- and 5-year data, respectively. Large ES, SRM and GRI ranging 2.66–2.78, 2.42–2.61 and 1.67–1.88 were noted for Mayo hip scores with THA, respectively. The MCII and moderate improvement thresholds were 22.4–22.7 and 39.4–40.5 respectively. Hazard ratios of revision surgery were higher with lower final score or less improvement in Mayo hip score at 2-years and borderline significant/non-significant at 5-years, respectively: (1) score ≤55 with hazard ratios of 2.24 (95 % CI, 1.45, 3.46; *p* = 0.0003) and 1.70 (95 % CI, 1.00, 2.92; *p* = 0.05) of implant revision subsequently, compared to 72-80 points; (2) no improvement or worsening score with hazard ratios 3.94 (95 % CI, 1.50, 10.30; *p* = 0.005) and 2.72 (95 % CI, 0.85,8.70; *p* = 0.09), compared to improvement >50-points. Mayo hip score had significant positive correlation with younger age, male gender, lower BMI, lower ASA class and lower Deyo-Charlson index (*p* ≤ 0.003 for each) and with Harris hip scores (*p* < 0.001).

**Conclusions:**

Mayo Hip Score is valid, sensitive to change and associated with future risk of revision surgery in patients with primary THA.

**Electronic supplementary material:**

The online version of this article (doi:10.1186/s12891-016-0868-3) contains supplementary material, which is available to authorized users.

## Background

Hip replacement has been termed the operation of the century [1]. Improvements after total hip arthroplasty (THA) are often measured using instruments that measure pain, function and/or quality of life (QOL). Patient-reported outcomes (PROs) of pain and QOL show improvement after THA, usually an elective procedure for the treatment of end-stage hip arthritis. A recent review of key challenges to the assessment of QOL outcomes in arthroplasty noted that it is critical to assess psychometric properties of commonly used instruments [2]. Most studies use composite instruments (pain, function and QOL) to assess arthroplasty outcomes, the most common being the Harris Hip Score (HHS) [3, 4]. The Mayo Hip Score, a composite instrument that assesses pain, function and hip mobility, has been used for THA outcome assessment at our institution (i.e., Mayo Clinic) for >3 decades. A key difference between the Mayo Hip Score and the HHS is that Mayo Hip Score is shorter and does not include range of motion and limb deformity assessments [5, 6]. Thus, it does not require a physician’s examination or a goniometer for completion and can be easily completed by a patient (or a provider).

Previous studies have provided initial evidence for construct validity and test-retest reliability of the Mayo Hip Score [5, 6], but other aspects of validity and discriminant ability have not been tested. It is important that the instruments used for THA outcomes assessment are valid, reliable and responsive to change. Responsiveness to change is an important attribute of a PRO instrument, since they are often used to compare outcomes of technological and surgical innovations in THA [7, 8]. The objective of this study was to examine discriminant ability, association with subsequent revision and examine further construct validity and reliability of the Mayo Hip Score.

## Methods

### Study participants and the instrument

The study included patients who had undergone primary THA at the Mayo Clinic and had completed a Mayo Hip Score at preoperative and/or one of the two follow-up periods, i.e. 2- or 5-years post-THA. The institutional review board at the Mayo Clinic approved the study and waived the requirement for patient consent. The Mayo Hip Score is a composite scale that consists of pain and function questions (Additional file 1: Appendix 1). The total score ranges 0–80; higher score is better. A single pain question is scored 0–40 points based on pain severity: no pain, slight, moderate and severe pain scored at 40, 35, 20 and 0 points. Hip function is based on distance walked (15 points) and the use of support aids (canes, crutches, walker; 5 points for no aids) for ambulation. Hip mobility is assessed with 4 questions, each for 5 points: ability to enter and leave a car, ability to perform foot care, presence of a limp and the ability to climb stairs. The Mayo Hip Score has been shown to have test-retest reliability and limited construct validity [5, 6]. The study period was 1993–2005.

### Responsiveness/discriminant validity, construct validity and association with revision

Responsiveness was assessed by three statistics. We calculated the effect size (ES) by dividing the change in hip score from baseline to 2-years by the standard deviation at baseline (preoperative). An ES of 0.20–0.49 represents a small change, 0.50–0.79 a medium change, and ≥ 0.80 a large change, according to Cohen’s rule. The standardized response mean (SRM) is defined as the mean change in the patient Mayo hip score divided by the SD of the changed scores. The Guyatt Responsiveness Index (GRI) is the ratio of average change in patients identified as improved (much better and somewhat better combined as one group) divided by the standard deviation of the change in patients identified as remaining stable (“no change”) based on the global rating of hip function change.

We calculated the minimal clinically important improvement (MCII) and moderate improvement as improvements corresponding to categories “somewhat better now” and “much better now”, on the following global question as the anchor: Compared to before surgery, how is your hip? Much better now, somewhat better now, the same and worse.

Construct validity was assessed with convergent and divergent validity. Convergent validity was assessed by Spearman’s correlation coefficients between Mayo hip scores and HHS at baseline, 2- and 5-years. We also assessed convergent validity by assessing Mayo hip scores for different activity levels (unlimited, some limitation and severe limitation). Divergent validity was assessed with examining mean Mayo hip scores at 2- and 5-years post-THA for the different levels of variables previously shown to be associated with pain/function outcomes after THA and examining association of these variables in univariate linear regression with Mayo hip scores [9–16]: age, gender, American Society of Anesthesiology (ASA) class and the Deyo-Charlson index [17], a validated measure of medical comorbidity. We also assessed association of Mayo hip scores with the number of joints involved (index hip or up to four joints including both knees and hips). These models examining were also subsequently adjusted for baseline Mayo Hip Scores. Test-retest reliability was assessed using intra-class coefficient (ICC) [18], by comparing two ratings on the same individual by patient vs. physician within 2-weeks of each other.

We examined the associations of the Mayo hip scores at 2- or 5-years or the change in Mayo hip scores from baseline to 2- and 5-years with the risk of subsequent revision THA. Final Mayo hip scores were categorized as ≤55, 56–63, 64–71 and 72–80, based on previously defined categories of excellent, good, fair and poor results [6]. Change in Mayo hip scores was categorized as ≤0, 1–25, 26–50 vs. >50. We used Cox regression model and calculated hazard ratios of revision, along with 95 % confidence intervals. We constructed Kaplan-Meier survival plots for absolute and change Mayo hip scores. A *p*-value of 0.05 or lower was considered significant.

## Results

Clinical and demographic features of study cohort are shown in Additional file 1: Appendix 2. Mean age was 64 years, 51 % were female. 24 % had body mass index (BMI) of <25 kg/m^2^ and mean Deyo-Charlson index was 1.0. Mean Mayo hip scores (standard deviation) at baseline pre-operative and 2- and 5-years post-THA were 33 (14), 72 (12) and 70 (13), respectively. Of all surveys available, 23–25 % each were for patients with pre-operative and 2-year and preoperative and 5-year assessment. Non-response rates at 2- and 5-years in those with preoperative surveys were 36 % and 45 %.

Discriminant validity/Responsiveness to Change: Large ES, SRM and GRI were noted for Mayo hip scores at both 2-years and 5-years post-THA (Table 1). MCII estimates were 22.4 at 2-years and 22.7 at 5-years; moderate improvement thresholds were 40.5 and 39.4, respectively (Table 1).

**Table 1:** Discriminant ability of Mayo hip score and thresholds for clinically meaningful improvements

Discriminant ability statistics		
	2-year - Baseline value	5-year - Baseline value
Effect Size (ES)	2.78 [*n* = 2,278]	2.66 [*n* = 2,089]
Standardized Response Mean (SRM)	2.61 [*n* = 2,050]	2.42 [*n* = 1,919]
Guyatt’s Responsiveness Index (GRI)	1.67 [*n* = 27]	1.88 [*n* = 37]
Clinically Important Improvement Thresholds, Mean (standard deviation)		
Minimally clinically important improvement (MCII)	22.4 (19.5) [*n* = 122]	22.7 (19.4) [*n* = 131]
Moderate improvement	40.5 (14.1) [*n* = 1,928]	39.4 (14.9) [*n* = 1,788]

Convergent and divergent validity: Younger age, male gender, lower BMI, lower ASA class and lower Deyo-Charlson index were each significantly associated with higher Mayo hip scores at 2- and 5-years post-THA (*p* ≤ 0.003 for each; Additional file 1: Appendix 3), while the total number of joints involved was not significantly associated. This finding was confirmed in models that additionally adjusted for baseline preoperative Mayo hip scores (Additional file 1: Appendix 3). Similar strong associations of Mayo hip scores were seen with lower levels of overall activity limitation and with patient response of more improvement in their hip (much better, better) on the global question (Additional file 1: Appendix 3). Spearman correlation coefficients of Mayo Hip Score with Harris Hip Score at baseline were 0.91 and 0.93, at 2-years and 5-years respectively (*p* < 0.001 for all).

Association with Subsequent Implant Revision: Mayo hip scores of 55 or lower at 2-years were significantly associated with higher risk of THA revision at 2-years (*p* = 0.0003) and with borderline significance at 5-years (*p* = 0.05), the risk being 2-times compared to those with score 72–80 points (Table 2). Compared to improvement of >50-points in Mayo hip score, patients with no improvement or worsening of Mayo hip score at 2-years after THA had a 3.9 times higher risk of THA revision subsequently (*p* = 0.005) and a statistically non-significant trend at 5-years with 2.7-times odds (*p* = 0.09; Table 2).

**Table 2:** The association of Mayo Hip Scores with the risk of THA revision using Cox regression analyses

	Hazards of THA Revision (95 % CI)	*p*-value	Hazards of THA Revision (95 % CI)	*p*-value
	2-year data		5-year data	
	*n* = 3,307 with 178 events		*n* = 1,717 with 97 events	
Total Mayo Hip Score^a^				
≤55 vs. 72–80	2.24 (1.45, 3.46)	0.0003	1.70 (1.00, 2.92)	0.05
56–63 vs. 72–80	0.60 (0.24, 1.48)	0.26	1.00 (0.40, 2.52)	0.99
64–71 vs. 72–80	0.80 (0.48, 1.36)	0.42	0.70 (0.35, 1.42)	0.32
	2-year data		5-year data	
	*n* = 1,904 with 106 events		*n* = 1,080 with 61 events	
Mayo Hip Score improvement				
≤0 vs. >50	3.94 (1.50, 10.30)	0.005	2.72 (0.85, 8.70)	0.09
1–25 vs. >50	0.88 (0.44, 1.76)	0.71	1.45 (0.70, 3.01)	0.32
26–50 vs. >50	0.87 (0.56, 1.34)	0.52	0.73 (0.40, 1.35)	0.32

^a^Categorization of Mayo hip score is based on the previously described categories: Excellent result is 72-80 points, good is 64–71 points, fair is 56–63 points, and poor is ≤55 points by McGrory et al. 1996 [6]

**Fig. 1:**
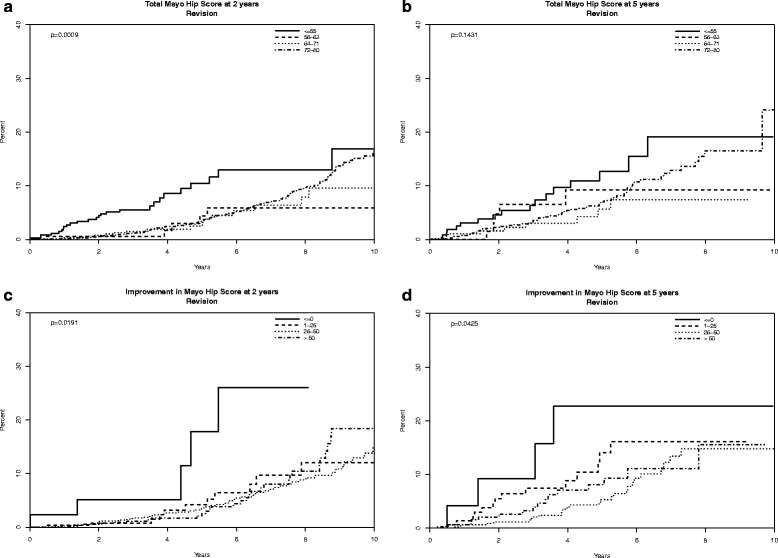
Implant failure (revision) graphs based on the absolute Mayo hip scores at 2-years and 5-years (panels a and b) and change in scores compared to preoperative (panels c and d) in the subsequent follow-up period. **a** Absolute Mayo hip scores at 2-years and subsequent revision risk. **b** Absolute Mayo hip scores at 5-years and subsequent revision risk. **c** Improvement in Mayo hip scores at 2-years and subsequent revision risk. d. Improvement in Mayo hip scores at 5-years and subsequent revision risk. X-axis represents the number of years after the survey completion (survey completion was 2-years post-THA for panels **a** and **c**; 5-years post-THA for panels **b** and **d**), and y-axis the proportion of patients who underwent revision surgery for the hip implant

The association of 2- and 5-year final and change scores on Mayo hip scores with implant survival is shown in Fig. 1. Absolute Mayo hip scores at 2-years and change in Mayo hip scores at 2- and 5-years were each significantly associated with the risk of revision (*p* < 0.05 each); absolute Mayo hip scores at 5-years showed a non-significant trend (*p* = 0.14).

Test-retest reliability: The Mayo hip scores from physician and patient-administered surveys were numerically similar, 73 +/- 10 vs. 68 +/- 12, being 5-point (SD, 10) higher in physician- than patient-assessed surveys. In contrast, for HHS, respective scores were 91 +/- 12 vs. 81 +/- 14 with a difference of 10 +/- 11 between the scores. The ICC for Mayo hip score, using the data from the physician vs. patient-administered, was high at 0.55 (95 % CI: 0.47, 0.63), numerically higher than the ICC for Harris Hip Score at 0.48 (95 % CI: 0.40, 0.57).

## Discussion

In this study, we found that Mayo hip score is a valid and reliable measure of hip outcomes in patients who had undergone primary THA. Mayo hip score was also associated with the risk of revision surgery at 2-years, both when examined as the final score and as a change score. These psychometric properties compared well to the HHS [5, 19–21], which at present is the most commonly used outcome instrument for THA outcome assessment [3, 4]. An advantage of the Mayo hip score is that either physicians or patients can complete it, as compared to the HHS that has physician and patient portions. An excellent agreement between these assessments also indicates that in an appropriate setting, Mayo hip score may be an alternative to HHS. Several findings from this validation study deserve further discussion.

First, our study established that Mayo hip score was responsive to change with large effect sizes and was associated with risk of subsequent revision at 2-years. This is a very important finding, considering that one of the main applications of arthroplasty outcomes instruments is to compare various surgical or implant types and therefore a responsive instrument is highly desirable [2]. HHS has been shown to be responsive with large ES and SRM of 2.5 and 1.8 [21]; respective ES and SRM for Mayo hip score of 2.8 and 2.6 from our study are comparable. We also determined the MCII and moderate improvement thresholds for Mayo hip score, at 2- and 5-years.

The association of Mayo hip score with the risk of future revision surgery is particularly impressive, since the attainment of Mayo Hip Scores at or below 55 points at 2- or 5-years increased the risk of early revision surgery after each time-point by 2-times, compared to scores 72–80 points (maximum score is 80). This was statistically significant at 2-years (*p* = 0.0003) and borderline statistically significant at 5-years (*p* = 0.05). A lack of improvement (or worsening) of Mayo hip score from preoperative to 2-years was associated with a 3-4 times higher revision risk compared to improvement by >50-points. K-M survival curves describe these findings that both absolute and change in Mayo hip scores were significantly associated with the risk of revision, the only exception being the association of absolute scores at 5-years that was not statistically significantly associated with future revision risk (*p* = 0.14). These findings indicate that Mayo hip scores might allow screening of early THA implant failures. This has practical implications, beyond instrument validation. It may be possible to develop an early implant risk score for clinical use that incorporates Mayo hip score (or similar scales) and other risk factors, a project currently underway.

Second, this study adds significant validation data related to the construct validity of the Mayo hip score. Our finding of significant association of Mayo hip scores at 2- and 5-years with important baseline characteristics previously shown to impact pain and function outcomes, i.e. age, gender, BMI, ASA class and Deyo-Charlson index [9–16], provides critical evidence for its convergent and divergent validity. The high correlation of Mayo hip score with HHS, a validated outcome instrument [19, 20, 22] and overall activity level, establishes the construct validity of Mayo hip score for assessment of outcomes after THA. More validation studies in other populations such as revision THA, hip arthroscopy, partial hip replacement may be needed before Mayo hip score can be used for the assessment of outcomes in these populations.

We found the Mayo hip score has fair test-retest inter-rater reliability, which extends the findings from an independent sample from our institution (i.e., Mayo Clinic) in 97 THA patients [6]. We found a slightly higher (better) score in physician- than patient-administered Mayo hip score, and the difference of 5-points in our study was slightly higher than the 1.8-point difference in the previous study. As expected, this difference was slightly smaller than that for HHS, given its complexity and inclusion of range of motion and deformity. This indicates the reliability of Mayo Hip score is as much as HHS or higher. Inter-rater ICCs in the range of 0.39 to 0.86 have been reported for outcome measures in pemphigus [23] with patient vs. physician scoring, similar to our ICC of 0.55 for Mayo hip score. A higher ICC would imply that patient and physician assessments agree closely; however, the ICC seems numerically better than the most commonly used instrument, the HHS, in our study. For a patient-reported measure such as Mayo hip score, it is not surprising to see differences in physician completed assessment vs. patient completed assessment. We recommend that this PRO be completed by patients, not physicians, and in studies where both physicians and patients have completed the survey, MHS scores may not be interchangeable.

Key advantages of Mayo hip score are that it has fewer questions than HHS and does not require physician assessment in the clinic. Therefore, it can be sent as a mailed survey and completed by patients. This can allow for a rapid and efficient assessment of not only the patients’ current pain/function status that allows screening for THA failures during the short and long-term arthroplasty follow-up. As suggested in earlier studies, the Mayo hip score can be coupled with radiographic score [5] for a more comprehensive assessment. The advent of telemedicine may allow for a virtual patient visit and follow-up (mailed survey and remote review of radiographs) that can replace the annual in-person clinician visit. In an era of health care cost reduction and a declining supply of arthroplasty surgeons [24], this approach might allow more cost-efficient arthroplasty follow-up and monitoring.

One must consider the study limitations while interpreting study findings. Non-response rates were 36 % and 45 % at 2- and 5-years, respectively, which could have potentially affected the study findings. We decided *a priori* not to impute values for missing data, since this was a validation study, and we wanted to only use real data. It is unclear to us, whether it strengthened or weakened study findings, since we are unaware of the impact of non-response on validation statistics. These findings may therefore be generalizable only to patients who regularly respond to post-arthroplasty surveys. The overall revision rate in the cohort is low, and we may have missed significant findings due to the rarity of this outcome.

So, what does this study add? There is already a large diversity of instruments being used in THA assessments [25], so why have another instrument? Several instrument in current use in arthroplasty have limited data on validity and reliability, and we provide assessment of validity and responsiveness of an instrument that has been in use in our Total Joint Registry for >3 decades and is patient self-administered. One challenge in post-arthroplasty outcome assessment is the use of several different outcome measures [25]. Harmonization of the THA outcome instrument use across arthroplasty studies would be a step forward and will allow comparison across studies. This effort is  currently underway [26].

## Conclusions

In summary, this study provides validation data for the Mayo hip score. Mayo hip score is valid, reliable and sensitive to change. Mayo hip score is associated with the risk of subsequent revision surgery. It correlates well with HHS, the most popular instrument for THA outcomes assessment and has the advantage that it can be completed either by the patient or the surgeon. We suggest that this instrument be completed by the patient, based on the fact that pain and daily function abilities are best assessed and reported by a patient and that it is more practical for the patient to complete this instrument than the physician. Clinically meaningful thresholds for Mayo Hip score have been established. In the era of where PROs are a critical part of patient assessment and follow-up after arthroplasty, the Mayo hip score may offer an alternative to longer instruments.

## Additional file

Additional file 1:
**Appendix 1**. Mayo Hip Score: Clinical Assessment Score (0-80 points). **Appendix 2**. Demographic and clinical characteristics of the study population. **Appendix 3**. Convergent and Divergent validity using unadjusted linear regression models and those adjusted for baseline Mayo hip scores. (DOCX 92 kb)

## Abbreviations

THA: Total hip arthroplasty
HHS: Harris hip score
QOL: Quality of life
ASA: American society of anesthesiology
BMI: Body mass index
PRO: Patient-reported outcomes
MCII: Minimal clinically important improvement
ASA: American society of anesthesiology
ES: Effect size
SRM: Standardized response mean
GRI: Guyatt’s responsiveness index
