# Supplementary material for: Validation of the Mayo Hip Score: construct validity, reliability and responsiveness to change
Source: BMC Musculoskelet Disord. 2016 Jan 19;17:39. doi: 10.1186/s12891-016-0868-3 (PMC4719668; doi:10.1186/s12891-016-0868-3)
Supplement: Additional file 1: — Appendix 1. Mayo Hip Score: Clinical Assessment Score (0-80 points). Appendix 2. Demographic and clinical characteristics of the study population. Appendix 3. Convergent and Divergent validity using unadjusted linear regression models and those adjusted for baseline Mayo hip scores. (DOCX 92 kb) [file 12891_2016_868_MOESM1_ESM.docx]

**Appendix**

**Appendix 1. Mayo Hip Score: Clinical Assessment Score (0-80 points)**

| **Clinical parameter** | **Points** |
| --- | --- |
| **Pain** |  |
| None | 40 |
| Slight or occasional | 35 |
| Moderate | 20 |
| Severe | 0 |
|  |  |
| **Function** |  |
| **Distance walked** |  |
| ≥10 blocks | 15 |
| 6 blocks | 12 |
| 1-3 blocks | 7 |
| Indoors | 2 |
| Unable to walk | 0 |
| **Support aids** |  |
| None | 5 |
| Occasional use of a cane | 4 |
| Full-time use of a cane/crutch | 3 |
| Two canes or crutches | 2 |
| Walker | 1 |
| Unable to walk | 0 |
| **Mobility and muscle power In and out of car** |  |
| With ease | 5 |
| With difficulty | 3 |
| Unable | 0 |
| **Foot care** |  |
| With ease | 5 |
| With difficulty | 3 |
| Unable | 0 |
| **Limp** |  |
| None | 5 |
| Slight | 3 |
| Severe | 0 |
| **Ability to climb stairs** |  |
| Normal | 5 |
| Holding rail | 4 |
| One step at a time | 2 |
| Unable | 0 |
|  |  |
|  |  |

**Appendix 2. Demographic and clinical characteristics of the study population**

|  | **Hips with Mayo hip scores at baseline (n=5,307)**  **N (%)** | **Hips with Mayo hip scores at baseline and 2 years (n=2,278)**  **N (%)** | **Hips with Mayo hip scores at baseline and 5 years (n=2,089)**  **N (%)** |
| --- | --- | --- | --- |
| **Age in years, Mean (SD)** | 64 (14) | 65(13) | 64 (13) |
| **Male** | 2563 (48.3%) | 1102 (48.4%) | 1007 (48.2%) |
| **Age Groups** |  |  |  |
| **≤60 years** | 1747 (32.9%) | 699 (30.7%) | 681 (32.6%) |
| **61-70 years** | 1583 (29.8%) | 731 (32.1%) | 680 (32.6%) |
| **71-80 years** | 1567 (29.5%) | 667 (29.3%) | 607 (29.1%) |
| **> 80 years** | 410 (7.7%) | 181 (8.0%) | 121 (5.8%) |
| **Body Mass Index^1^** |  |  |  |
| **<25 kg/m^2^** | 1278 (24.2%) | 546 (24.1%) | 486 (23.4%) |
| **25-29.9 kg/m^2^** | 2033 (38.5%) | 895 (39.4%) | 821 (39.5%) |
| **30-34.9 kg/m^2^** | 1281 (24.2%) | 546 (24.1%) | 507 (24.4%) |
| **35-39.9 kg/m^2^** | 445 (8.4%) | 186 (8.2%) | 179 (8.6%) |
| **≥ 40 kg/m^2^** | 249 (4.7%) | 96 (4.2%) | 85 (4.1%) |
| **American Society of Anesthesiologists**  **Class (ASA)^2^** |  |  |  |
| **Class I-II** | 3279 (62.0%) | 1428 (63.0%) | 1348 (64.8%) |
| **Class II-IV** | 2006 (38.0%) | 838 (37.0%) | 733 (35.2%) |
| **Deyo-Charlson Index, mean (SD)** | 1.0 (1.9) | 1.0 (1.8) | 0.9 (1.7) |
| **Psychological Comorbidity** |  |  |  |
| **Anxiety** | 241 (4.5%) | 93 (4.1%) | 91 (4.4%) |
| **Depression** | 377 (7.1%) | 139 (6.1%) | 131 (6.3%) |
| **Number of Joints Replaced** |  |  |  |
| **1** | 4357 (82.1%) | 1836 (80.6%) | 1682 (80.5%) |
| **2** | 875 (16.5%) | 410 (18.0%) | 376 (18.0%) |
| **3** | 65 (1.2%) | 27 (1.2%) | 25 (1.2%) |
| **4** | 10 (0.2%) | 5 (0.2%) | 6 (0.3%) |
| **Underlying Diagnosis** |  |  |  |
| **Osteoarthritis** | 4595 (86.6%) | 2011 (88.3%) | 1836 (87.9%) |
| **RA/Inflammatory Arthritis** | 137 (2.6%) | 57 (2.5%) | 49 (2.4%) |
| **Other** | 575 (10.8%) | 210 (9.2%) | 204 (9.8%) |

Missing data at preoperative, preoperative and 2-years and preoperative and 5-years cohorts were as follow: ^1^ BMI data: 21, 9 and 11 patients respectively;

^2^ ASA data: 22, 12 and 8 patients respectively

**Appendix 3. Convergent and Divergent validity using unadjusted linear regression models and those adjusted for baseline Mayo hip scores**

| **Variables of interest** | **Outcomes** | | | |
| --- | --- | --- | --- | --- |
|  | **Mayo Hip Score- 2 years** | | **Mayo Hip Score- 5 years** | |
|  | Estimate (standard error) | p-value | Estimate (standard error) | p-value |
| **Unadjusted Models** | | | | |
| Age per 10 years | **-1.20 (0.17)** | **<0.001** | **-1.46 (0.20)** | **<0.001** |
|  |  |  |  |  |
| Male | **2.49 (0.45)** | **<0.001** | **2.66 (0.51)** | **<0.001** |
|  |  |  |  |  |
| # of joints | -0.22 (0.50) | 0.65 | -0.10 (0.55) | 0.85 |
|  |  |  |  |  |
| BMI | **-0.26 (0.04)** | **<0.001** | **-0.27 (0.05)** | **<0.001** |
|  |  |  |  |  |
| ASA class 3 or 4 | **-3.33 (0.46)** | **<0.001** | **-4.15 (0.53)** | **<0.001** |
|  |  |  |  |  |
| Deyo-Charlson Index | **-0.46 (0.12)** | **<0.001** | **-0.43 (0.15)** | **0.003** |
|  |  |  |  |  |
| Activity |  |  |  |  |
| Mild limitation^1^ | **19.37 (0.90)** | **<0.001** | **15.54 (0.80)** | **<0.001** |
| Unlimited^1^ | **27.04 (0.90)** | **<0.001** | **24.9 (0.80)** | **<0.001** |
|  |  |  |  |  |
| Overall Response |  |  |  |  |
| Better^2^ | **5.49 (1.45)** | **<0.001** | **10.61 (1.44)** | **<0.001** |
| Much better^2^ | **26.87 (1.25)** | **<0.001** | **29.99 (1.21)** | **<0.001** |
| **Models adjusted for Baseline Mayo Hip Scores** | | | | |
| Age per 10 years | **-1.02 (0.18)** | **<0.001** | **-1.24 (0.21)** | **<0.001** |
| Baseline^3^ | **0.15 (0.02)** | **<0.001** | **0.15 (0.02)** | **<0.001** |
|  |  |  |  |  |
| Male | **2.07 (0.47)** | **<0.001** | **2.37 (0.54)** | **<0.001** |
| Baseline^3^ | **0.15 (0.02)** | **<0.001** | **0.15 (0.02)** | **<0.001** |
|  |  |  |  |  |
| # of joints | -0.45 (0.52) | 0.37 | -0.54 (0.59) | 0.36 |
| Baseline^3^ | **0.16 (0.02)** | **<0.001** | **0.16 (0.02)** | **<0.001** |
|  |  |  |  |  |
| BMI | **-0.21 (0.04)** | **<0.001** | **-0.23 (0.05)** | **<0.001** |
| Baseline^3^ | **0.15 (0.02)** | **<0.001** | **0.15 (0.02)** | **<0.001** |
|  |  |  |  |  |
| ASA 3, 4 | **-2.53 (0.49)** | **<0.001** | **-3.53 (0.57)** | **<0.001** |
| Baseline^3^ | **0.15 (0.02)** | **<0.001** | **0.14 (0.02)** | **<0.001** |
|  |  |  |  |  |
| Charlson Index | **-0.38 (0.13)** | **0.003** | **-0.42 (0.16)** | **0.007** |
| Baseline^3^ | **0.16 (0.02)** | **<0.001** | **0.15 (0.02)** | **<0.001** |
|  |  |  |  |  |
| Activity |  |  |  |  |
| Mild limitation^1^ | **17.85 (0.96)** | **<0.001** | **14.67 (0.88)** | **<0.001** |
| Unlimited^1^ | **25.10 (0.97)** | **<0.001** | **23.74 (0.90)** | **<0.001** |
| Baseline^3^ | **0.07 (0.02)** | **<0.001** | **0.05 (0.02)** | **0.005** |
|  |  |  |  |  |
| Overall Response |  |  |  |  |
| Better^2^ | **4.22 (1.51)** | **0.005** | **9.50 (1.52)** | **<0.001** |
| Much better^2^ | **25.56 (1.30)** | **<0.001** | **28.51 (1.27)** | **<0.001** |
| Baseline^3^ | **0.13 (0.01)** | **<0.001** | **0.14 (0.02)** | **<0.001** |

^1^ reference is limited activity

^2^ reference is no improvement

^3^ Baseline indicates adjustment for the preoperative Mayo hip score

Shaded bar separates each univariate and multivariable-adjusted model; Numbers in bold represent statistically significant association.

The models with the baseline variables have fewer observations than those without the baseline variables, i.e. the number of observations for Mayo Hip Score decreases from 2657 to 2228 at 2-years and from 2435 to 2089 at 5-years.
